# Post-Testicular Sperm Maturation: Centriole Pairs, Found in Upper Epididymis, are Destroyed Prior to Sperm’s Release at Ejaculation

**DOI:** 10.1038/srep31816

**Published:** 2016-08-18

**Authors:** C. Simerly, C. Castro, C. Hartnett, C. C. Lin, M. Sukhwani, K. Orwig, G. Schatten

**Affiliations:** 1 Department of Obstetrics, Gynecology and Reproductive Sciences, 300 Halket Street, University of Pittsburgh School of Medicine, Pittsburgh, PA 15213, USA; 2 Department of Cell Biology and Physiology, 3500 Terrace Street, University of Pittsburgh School of Medicine, Pittsburgh, PA 15213, USA; 3 Magee-Womens Research Institute and Foundation, Pittsburgh, 204 Craft Avenue, PA 15213, USA; 4 Department of Bioengineering, University of Pittsburgh, 300 Technology Drive, Pittsburgh PA 15213, USA.

## Abstract

The fertilizing sperm’s lengthiest unchartered voyage is through the longest, least-investigated organ in a man’s body – the Epididymis. Over six meters long in men, ~80 meters in stallions and over one-hundred times a mouse’s body length, there are few functions known aside from sperm storage and nutrition. While spermatogenesis is completed in the testes, here we demonstrate sperm centriole reduction occurs within the epididymis. Investigations of GFP-CENTR mice and controls demonstrate both the presence of centriole pairs in the upper caput region of the epididymis and, the destruction, first, of the distal and, then, of the proximal centriole as the sperm transits to the cauda and vas deferens in preparation for its climactic release. These centrioles can neither recruit γ-tubulin nor nucleate microtubules when eggs are inseminated or microinjected, yet numerous maternally-nucleated cytasters are found. These sperm centrioles appear as vestigial basal bodies, destroyed in the mid-to-lower corpus. Post-testicular sperm maturation, in which sperm centrioles found in the caput are destroyed prior to ejaculation, is a newly discovered function for the epididymis.

The purpose of the epididymis^1^,^2^,^3^,^4^,^5^ remains mysterious and the reasons for the sperm’s extensive journey is perplexing. If a sperm were human-sized, its week or two trek through the epididymis would be ~2,750 kilometers^6^. Testicular sperm, and those collected from the upper epididymal regions from both mice and men are competent for reproduction when injected using intracytoplasmic sperm injection (ICSI), demonstrating their reproductive competence^7^,^8^. With advances in assisted reproductive technologies (ART), men without any sperm in their ejaculates are able to conceive through the application of sophisticated sperm-retrieval protocols^9^ and ICSI^10^ from epididymal sources. ART success rates are higher with epididymal sperm than with testicular ones, suggesting post-testicular maturation^11^. Noteworthy investigations using primarily bull or mouse epididymal isolates have found important regional differences in gene expression patterns^12^, proteins^13^,^14^, and post-translational modifications^15^, as well as epididymosomes^4^, important vesicles found in the epididymal lumen. Here we show sperm maturation occurring within the epididymis involves a previously unappreciated event. The sperm centriole-pair persist with the sperm after release into the lumen of testicular tubules and as they travel into the epididymis head. Nearly all sperm have centrioles in the caput, and first the distal and then the proximal centrioles are destroyed as they pass through the corpus to reach the cauda epididymis on their transit to the vas deferens. The destruction of these centrioles is neither accelerated in young males nor slowed in older ones, though individual variabilities are found. Further, caput sperm with intact centriole pairs are unable to nucleate microtubules when introduced into metaphase-II oocytes. The term ‘Zombie centrioles’ recently introduced by Khire *et al*.^16^, seems apt to describe these non-functional centrioles awaiting their disassembly in the male reproductive tract.

The sperm is thought to contribute primarily the imprinted haploid genome and the sperm centrosome to the offspring. The sperm tail^17^ and the sperm mitochondria^18^,^19^ are destroyed as the zygote develops, though the fate of the individual molecules has not been tracked. RNA has been found in sperm^20^,^21^ and diminished RNA content correlates with idiopathic male infertility^22^, suggesting that RNA may be vital for early embryonic development, and, most intriguingly, male epigenetic transmission^23^,^24^.

Centrosomes^25^,^26^,^27^,^28^ are usually contributed by the sperm during fertilization^29^,^30^, and, after recruitment of maternal microtubule organizing center [MTOC] components^31^, the functional mitotic spindle poles are reconstructed each generation for embryonic cell divisions. Like the chromosomes, which are reduced in half during meiosis, centrosomes must also be reduced during gametogenesis^16^, such that the proper number is restored in the zygote. During female meiosis, centrioles are destroyed and centrosomes disaggregate in the maturing oocyte. However in males, neither centrosome reduction mechanisms nor sperm centriole fate is well understood. Additionally, mice seem to be nearly unique, at least in comparison to non-rodent mammals, in having maternal inheritance of the centrosome or MTOC^32^,^33^,^34^. Convincing studies demonstrate the absence of centrioles and centrosomes in mouse cauda epididymal sperm, consistent with the accepted view that, unlike other animals, paternal centriole and centrosome inheritance does not occur in rodents^35^,^36^.

Past studies with anticentrin antibody 20H5 and transmission electron microscopy (TEM) detected distal and proximal centrioles in isolated testicular round and elongating spermatids, with reported centrin antibody reduction beginning after spermiation and complete loss in mature caput sperm^37^. From these observations, then, it was surprising to still detect sperm centrioles in the epididymis of GFP-centrin transgenic mice (Figs 1 and 2). Direct imaging (Fig. 1A) and immunocytochemistry (Fig. 1B) of transgenic CB6F1 sperm expressing GFP-CETN2 demonstrates a pair of centrioles in the implantation fossa region at the base of the sperm head. γ-tubulin^38^, the rare tubulin isoform essential for microtubule nucleation, surrounds the centrioles in caput sperm (Fig. 1C). The presence of these centrioles has been confirmed in control, non-transgenic CB6F1 mice, using specific molecular probes; e.g., Fig. 1E,F shows sperm centriole pairs at the implantation fossa in non-transgenic mice detected with centrin monospecific antibodies. ~97% of sperm in the caput display centrioles, whereas only 5.4% and 2.5% of sperm have centrin-containing centrioles in the cauda and vas deferens, respectively (Figs 2 and 3). Identical findings for centriole detection and dissolution during epididymal transport were confirmed in GFP-expressing DBA and Nude/+ mouse spermatozoa isolated from the caput, corpus, and cauda epididymis (Supp. Fig. 4) and immunostained with anti-centrin 20H5, indicating that these observations in GFP-CETN2 males were not confined to this specific mouse strain.

Analysis of direct expression of GFP CETN2 in a cross section of a testis tubule from the basement membrane (Supp. Fig. 2B: *) to the lumen (Supp. Fig. 2B: #) extended these findings to include centriole detection in spermatogonia and primary spermatocytes aligned adjacent to the basement membrane (Supp. Fig. 2C), loss of GFP CETN2 in distal round spermatids (Supp. Fig. 2D), and reappearance of GFP CETN2 in elongating spermatids prior to their release into the lumen (Supp. Fig. 2E). Loss of GFP expression during the round spermatid stage was surprising, giving previous TEM studies showing the presence of normal centrosomes (a pair of centrioles and γ-tubulin)^36^. However, round spermatids do not nucleate microtubules and it was curious that this loss of microtubule organization ability coincides with the loss of GFP-CETN2 expression (Supp. Fig. 2D). Perhaps centriole alterations occur in the round spermatid stage as they switch from radial microtubule nucleating centers during meiosis to flagella organizing centers in the developing sperm tail in early elongating spermatids, creating a disruption in GFP CETN2 expression. At the beginning of sperm elongation, GFP-CETN2 expression returns as the sperm head is reshaped and the axoneme is elongating (Supp. Fig. 2E). Conversely, γ-Tubulin detection was observed in all testis cells including round and elongating spermatids (Supp. Fig. 2F–I, red) and both GFP CETN2 and γ-tubulin were prominent in meiotic spermatocytes (Supp. Fig. 2J–K). Likewise, direct GFP CETN2 expression was found in testicular sperm released into the rete testis (Supp. Fig. 3A,B) and transported into the efferent ducts (Supp. Fig. 3C–G) for transition into the initial segment of the caput epididymis.

Testicular sperm (Fig. 3) display both the proximal and distal centrioles, with the majority of γ-tubulin entering in the cytoplasmic droplet^39^ (Fig. 3A), itself destined to be sloughed off and discarded. Caput spermatozoa have strong proximal and distal centriole signals with centrin 20H5 (Fig. 3B), while the remaining γ-tubulin coalesces adjacent to the centriole pairs at the sperm axoneme’s basal body region. Sperm show reduced intensity for centrin (Fig. 3C,D) though γ-tubulin detection remains prominent (red, arrows) during epididymal transit. In sperm from the lower corpus (Fig. 3E), cauda (Fig. 3F), and vas deferens (Fig. 3G), centrin is no longer detectable at either centriole though most sperm retain detectable residual γ-tubulin.

To determine the function of these centrioles in epididymal sperm, we investigated whether sperm would nucleate astral microtubules in mature mouse oocytes following ICSI^40^,^41^ (Fig. 4) or sperm- zona-free oocyte fusion (Fig. 5). During early sperm insemination, major functions of acquired centrioles are to organized pericentriolar material (i.e. γ–tubulin) and assemble microtubules in a sperm aster responsible for initiating genome apposition in the activated cytoplasm^29^. Contrary to expectations, epididymal sperm centrioles neither recruit the oocyte’s γ-tubulin nor participate in any cytoplasmic event, though numerous ooplasmic cytasters are found^42^,^43^. Figure 4 demonstrates that the sperm centrioles from the caput are non-functional after ICSI. Figure 4A shows GFP-CETN2 at the centriole pair of a caput spermatozoon one hour after ICSI. Microtubules are not detected assembling at the sperm head as might be predicted if a sperm aster were to form. The sperm tail remains detectable, attached to the still condensed sperm head. The oocyte remains arrested at metaphase-II, and cytoplasmic microtubules, assembled into small cytasters, are found throughout the cytoplasm (red, short arrows). By 1.5–2 hrs post-ICSI (Fig. 4B), the microinjected sperm head decondenses, forming the male and female pronuclei as the second polar body is extruded. The GFP-CETN2 centrioles remain at the implantation fossa (Fig. 4B green, arrowheads) in a region devoid of assembling microtubules, even though many cytoplasmic MTOCs nucleate microtubules. In Fig. 4C,D, numerous microtubule asters assemble from cytoplasmic MTOCs at 3 hrs post-ICSI (D, E: red; microtubules, short arrows). Though one is near the male pronucleus (D, E: red, short double arrows), microtubules do not emanate from the GFP-CETN2 centrioles (D, E: green, arrowheads).

Zona-free oocytes fuse with ionomycin-activated caput sperm, which typically are unable to participate in fertilization until they reach the cauda^4^, but only in the presence of Sendai virus fusion extract. Motility normally is initiated only when they reach the cauda^44^. These zona-free oocytes are typically polyspermic (Fig. 5). However, neither caput nor cauda sperm centrioles nucleate microtubules following sperm incorporation (A–H). Both monospermic (Fig. 5A) and dispermic fertilizations (Fig. 5E) are shown at 6.5 hrs post-insemination. The sperm heads decondense with GFP-CETN2 centrioles detectable (Fig. 5A,E: green, arrowheads). Microtubules were not found to assemble adjacent to the incorporated sperm (A, E: left insets: red, microtubules). Oocytes remain at metaphase-II arrest (A, E: red), suggesting caput sperm, even after ionomycin, cannot initiate oocyte activation. These results suggest that sperm centriole reduction occurs after testicular release, with centriole pairs remaining in the upper epididymis prior to their destruction as they traffic through the epididymis. These sperm centrioles are transient, non-functional and appear incapable of duplicating. It is tempting to speculate that they are basal bodies which were essential for nucleating the sperm’s tail. Further, the destruction of these vestigial basal bodies might be required for the ultimate disintegration of the sperm tail after incorporation into the zygote^17^.

Since one of the roles of the epididymis is sperm storage, the time of sperm centriole destruction might be a result of either the age of the sperm or the male’s age. Sperm in the corpus and cauda are older than those in the caput, so studies to investigate GFP-CETN2 persistence in younger and older males were performed. By studying immature males as they initiate spermatogenesis, sperm have been examined as they first appear in the epididymis, following the ‘First wave of spermatogenesis^45^, i.e., just as they reach sexual maturity, the first sperm to enter the male reproductive tract. Barely detectable GFP centrin centrioles are found in the lower corpus epididymal tubules at 1.5- and 6-month-old males (Supp. Fig. 5A,B: green), with a significantly higher detection of centrin-labeled centrioles at 9 months (Supp. Fig. 5C). In cauda epididymis, the vast majority of spermatozoa have lost the GFP CETN2 expression at the implantation fossa. In the vas deferens, GFP CETN2 expression in spermatozoa at all ages is vastly reduced (A, B, C: green; insets, details; D: graph), though still statistically different at 9 months (D: graph, green bar; *: significantly different from 1.5 month male). Collectively, some males appear less efficient at centriole dissolution with advancing age. Aged males continue to exhibit the expected patterns of centrioles found in the caput and their destruction in the mid-to-lower corpus and cauda (Supp. Fig. 6) though aged males born in the same litter demonstrate variability. These results are consistent with clinical findings that there is considerable variability in male fertility during aging^46^. Sperm quality and semen production appear also to be of value for predicting age-related comorbidities in men^47^. These results suggest that neither age nor storage is rate-limiting factors.

*In vitro* modeling would be an important assay for understanding the mechanisms of centrosome and centriole reduction during spermatogenesis specifically, and perhaps centrosome-centriole disassembly generally. While worthwhile, developing this assay is challenging since the GFP-centrin signal is mostly lost after overnight culture. To explore the factors responsible for the destruction of sperm centriole loss *in vivo* as the sperm transits from the caput through the corpus and into the cauda epididymis, several experiments were performed. The assays compared centriole numbers in sperm isolated from caput, corpus and cauda regions. These were treated with the following: elevated external calcium or zinc ions with and without calcium ionophore, *in vitro* cultured epididymal cell lysates, and caput epididymosomes. Because none of these treatments alone or combined accelerated centriole destruction, it is tempting to speculate that the epididymal sperm already contain the seeds of their own centriole destruction.

Centrosome and centriole reductions are not well understood yet vital for development. In *Drosophila* sperm, centrosome reduction requires in part the loss of Asterless, a centrosomal protein regulated by polo-like kinase 4 [PLK4] and the ubiquitin kinase Slimb^16^. During *C. elegans* oogenesis, centrosome elimination requires cki-2, a cyclin-dependent kinase [cdk] inhibitor^48^. If destruction mirrors, in reverse order, centrosome and centriole construction, then the majority of γ-tubulin, NuMA and pericentrin may be discarded into the cytoplasmic droplet first. Later, the last of centriole core components, including the destruction of PLK4 and Asterless/CEP152 might be predicted to occur as in other animals, including perhaps humans and other mammals^29^,^49^, though species differences are anticipated. Centrosome and centriole reduction appears linked to the formation and shedding of the cytoplasmic droplet^50^,^51^, which often is extruding in the epididymis in many species. Furthermore, since complete centriole maturation requires two cell cycles^52^, perhaps the reverse process entailing the two meiotic cycles during spermatogenesis plays a role in eventual centriole dissolution in the epididymis.

Sperm, as they are generated in and released from the testis and enter the upper (caput) epididymis, are fertilization-incompetent. They then progress into the epididymal body (corpus) and, later, when they have reached the end of the epididymis (cauda), have acquired both motility and the ability to fertilize eggs. Remarkably, although nearly all sperm in the upper epididymis have centrioles (96.6 ± 0.05%), these have all been destroyed by the time the sperm have reached the end of the epididymis and are ready for their climactic release. Mature sperm are often described as those which have been released from the testicle. Complete functional spermatogenesis *in vitro* has been reported^8^, though the sperm generated were immotile and had to be microinjected into the oocytes.

These findings demonstrate a new form of ‘post-testicular sperm maturation’ or ‘epididymal maturation’^53^ in which sperm centrioles persist in the upper regions of the epididymis only to be destroyed prior to the sperm’s exit; these findings reveal previously unknown functions for this lengthy and still mysterious organ, which are essential for successful development, perhaps enabling innovative therapeutic targeting with the emerging centriole inhibitors for male contraceptive design^54^, and with defects likely responsible for idiopathic infertility or embryonic failures. Defects in human sperm aster assembly, possibly due to poor centrosome reconstitution following insemination, have been shown to lead to novel forms of human infertility^30^ and understanding the mechanism of mouse centriole dissolution perhaps raises potential strategies for discovering and designing novel methods to prevent human contraception. Recent evidence has now demonstrated that male epigenetic imprinting, especially the transmission of tRNA fragments, occurs in the epididymis as another example of ‘post-testicular sperm maturation’. Here we show that, concomitant with these imprinting events, the two sperm centrioles are lost with the distal ones destruction preceding the proximal centriole’s loss.

Epigenetic mechanisms, especially in the male germ line are now being eluciated^55^. Recently two publications^23^,^24^, reported discoveries on male epigenetic mechanisms involving transfer RNA fragments – with surprising evidence that the fragments are transmitted to the sperm well after sperm production, i.e. in the epididymis, long thought to be just an organ for sperm storage. These findings elevate the importance of epididymal maturation of sperm, i.e. ‘*Post-Testicular Maturation’,* to an entirely new level and force questions as to whether ‘*the epididymis is the epicenter for male epigenesis*’.

The epididymis, perhaps the least well understood of a man’s organs, and certainly the one which demands the greatest journey for his sperm, may finally be amenable for mechanistic investigations – with important implications for post-testicular epigenetic and centrosome maturations. These functions, in addition to those previously discovered, including management of reactive oxygen stress^13^, suggests that the epididymis is not only fundamental for fertilization and reproduction, but also the site at which vital modification essential for later development and transgenerational inheritance are instilled for the next and subsequent generations. If confirmed and extended, then the influence of environmental and behavioral assaults terminating in the epididymal transmission of the epigenetic information is a new field of vital importance for health and biology.

## Methods and Materials

### Animals, mouse husbandry and handling

All research was conducted according to the National Institute of Health’s Office of Laboratory Animal Welfare (OLAW) guidelines as described in *Guide for the Care and Use of Laboratory Animals* regulations. All ethics were reviewed and the research conducted with the approvals of our host Institutions, the University of Pittsburgh and Magee-Womens Research Institutional Animal Care and Usage Committees (IACUC; protocols #’s 13010894 and 14033317). Mice were purchased from approved commercial suppliers and housed in an AAALAC-accredited dedicated mouse facility (light cycle: 12 hr:12 hr). CB6-Tg (CAG-EGFP/CETN2)3-4Jgg/J mice (Jackson Laboratory, Bar Harbor, ME; Stock number: 008234; Higginbotham *et al*.^56^) were derived from cryopreserved stocks at the Jackson laboratory and hemizygous or wildtype genotypes acquired as juveniles. Mice expressing enhanced green fluorescent protein (GFP)-labeled human centrin-2 transgene were established by breeding 6- to 8-week-old hemizygous males or females with non-transgenic siblings or CB6F1/J inbred mice (Stock number: 100007; Jackson Labs). Transgenic mice born expressing GFP-centrin2 were identified by *PCR* or direct immunofluorescence, using tail-tip tissue (Supp. Fig. 1). Non-transgenic sibling CB6F1 and outbred CD-1 mice (Charles River, Raleigh, NC) were utilized for sperm experimental controls and oocyte collections to perform fertilization by ICSI or Sendai extract fusions. Epididymal tissues of GFP-DBA, produced for the Orwig laboratory by the MWRI Genome Editing, Transgenic and Virus Core (GETV, http://www.mwrif.org/125), and Nude/+ males (3–6 months post birth; Jackson Laboratory) were acquired from culled retired breeders at our host institute after sacrifice.

### Direct immunofluorescence and PCR for determining mice expressing the GFP CETN2 transgene

Detection of the GFP transgene in mouse pups bred from GFP CETN2 matings was accomplished by observing direct GFP fluorescence in tail tip tissues under conventional epifluorescence illumination and/or analyzing tail-tip tissue by *PCR* with MyTaq Extract-PCR Kit (Bioline, Taunton, MA). A 300-bp segment of the gene was amplified using the primers: 5′CCT ACG GCG TGC AGT GCT TCA GC 3′ (forward), 5′CGG CGA GCT GCA CGC TGC GTC CTC3′ (reverse). Β-actin served as a loading control, using the primers: 5′GAT GAC GAT ATC GCT GCG CTG GTC G3′ (forward), and 5′GCC TGT GGT ACG ACC AGA GGC ATA CAG3′ (reverse). *PCR* conditions were: 95 °C for 3 minutes, followed by 35 cycles of 95 °C for 15 seconds, 60 °C for 15 seconds, and 72 °C for 20 seconds. PCR product was analyzed in 2% agarose gel and visualized using ethidium bromide (Supp. Fig. 1).

### Testicular, epididymal, and vas deferens sperm collection

Reproductive tracts from GFP-CETN2-expressing males were collected intact into warm PBS after sacrificed by IACUC-approved protocols. After fat pad and mesenchymal tissues removal, the reproductive tracts were dissected into sections (testis, caput, upper corpus, middle upper corpus, mid-corpus, lower corpus, cauda, vas deferens) and placed individually into modified Human Tubal Fluid (HTF) medium without calcium or protein supplementation [mHTF-Hepes: 97.8 mM NaCl/4.69 mM KCl/0.20 mM MgSO_4_▪7H_2_O/0.37 mM KH_2_PO_4_/4.0 mM NaHCO_3_/21.0 mM HEPES/2.78 mM glucose/21.4 mM sodium lactate/100 U/ml penicillin/100 μg/ml streptomycin/5 mg/liter phenol red; formulation modified from Irvine Scientific, Santa Ana, CA] kept at 37°. Testicular cells and epididymal sperm were extruded with fine forceps or a sterile 30-guage needle, with care taken to avoid sample mixing. Testicular cells and sperm suspensions were passed through sterile 40-μm nylon mesh filters (Fisher Scientific, Pittsburgh, PA) to remove large cellular debris, centrifuged at 400 × g for 10 minutes at room temperature, and pellets suspended in fresh warm mHTF-Hepes at 37 °C until use.

### Oocyte collection and intracytoplasmic sperm injection (ICSI)

Non-transgenic CB6F1 or outbred CD-1 females (6–10 weeks) were superstimulated by intraperitoneal injection (i.p.) of 7.5 IU pregnant mares’ serum gonadotropin (Sigma-Aldrich, St. Louis, MO), followed 48 hrs later with an i.p. injection of 5.0 IU human chorionic gonadotropin (hCG; Sigma-Aldrich). Females were sacrificed beginning at 13 hrs post-hCG and ampullae excised into warm (37 °C) sterile EmbryoMax M-2 culture medium (EMD Millipore, Billerica, MA). Cumulus cells were removed with 0.5% hyaluronidase and mechanical pipetting. Washed, stripped oocytes were pooled in 100-μl droplets of M-2 under warm mineral oil (Irvine Scientific, Santa Ana, CA). ICSI was performed from the modified protocols of Stein and Schultz^41^, using a Nikon TE2000 inverted microscope equipped with Hoffman modulation contrast optics, an Eppendorf manipulator for a holding pipet, and a PrimeTech piezo drill (model PMAS-CT150; Sutter Instruments, Novato, CA) for sperm injection. Siliconized, 35°-angled, blunt-ended pipets, with an internal diameter of 5–6 μm, were prepared with a microforge, and backfilled with mercury prior to use. Isolated GFP-CETN2-expressing caput spermatozoa were enriched by centrifugation through a 1-ml 40% PureCeption column (Sage *in-vitro* Fertilization, Inc; Trumbull, CT) at 500 × g for 20 min, diluted into M-2 with 1% polyvinylpyrrolidone (Irvine Scientific), and the intact sperm with tails microinjected into mature oocytes. Inseminated oocytes were cultured in EmbryoMax KSOM culture medium in a 5% CO_2_ incubator until fixation.

### Zona-free *in vitro* insemination using Sendai extract

We utilized an inactivated Sendai virus cell fusion kit (HVJ Envelope: HVJ-E; Cosmo Bio, Tokyo, Japan) to fuse GFP-CETN2-expressing, ionomycin-treated caput epididymal sperm with zona-free oocytes, prepared by a 35–45 sec treatment with warm EmbryoMax acidic Tyrode’s culture medium (EMD Millipore). PureCeption^®^ enriched GFP-CETN2-expressing caput spermatozoa pre-incubated in HTF culture medium for 1 hr at 37 °C was treated with 10 μM ionomycin for 5 min, pelleted at 250 × g for 5 min, and reconstituted in HTF medium to ~220,000 sperm per ml. The immotile ionomycin-treated caput sperm were layered on top of zona-free oocytes in individual 15-μl droplets of HTF medium under oil and incubated for 1 hr to permit sperm-oocyte adherence. Oocytes with adhering sperm were transferred to 25 μl of ice-cold 1x fusion buffer containing a 1:25 dilution of Sendai HVJ-E extract in a sterile 35-mm glass-bottom dish (P35G-1.5-14-c; MatTek, Ashland, MA) on ice for 5 min and fusion initiated by gently flooding with 500 μl of warm 1x fusion buffer at 37 °C for 15 min. After incubation, ~1 ml of warm 1x fusion buffer containing 15 mg/ml BSA was added to detach oocytes from glass surfaces and each oocyte transferred individually to 15-μl droplets of HTF medium under mineral oil. Control IVF was performed by adding capacitated cauda epididymal sperm (~630,000 sperm/ml) to zona-free oocytes in HTF. All groups were incubated for 4–6.5 hrs at 37 °C in 5% CO_2_ in HTF until fixation.

### Immunocytochemistry

GFP CETN2 testicular cells and spermatozoa were attached to polylysine-coated (2 mg/ml; Sigma-Aldrich) 22 mm^2^ coverslips and fixed in 2% paraformaldehyde in mHTF-Hepes without calcium or protein supplementation for 10 minutes at 37 °C. Fixed specimens were rinsed in PBS with 0.25% Triton X-100 detergent (PBS-Tx), blocked 30 min at room temperature in a BlockAid (ThermoFisher Scientific, Grand Island, NY), and primary antibodies applied simultaneously overnight at 4 °C. Primary antibodies utilized included: mouse anti-centrin 20H5 (1:200; EMD Millipore), rabbit Ak15 anti-γ-tubulin (Sigma-Aldrich; 1:500), mouse acetylated α-tubulin 6–11B-1 (1:100; Santa Cruz Biotechnology, Dallas, TX), and rat anti-tubulin YOL 1/34 (1:200; EMD Millipore). After excess primary antibody was removed with PBS-Tx, appropriate fluorescently tagged secondary antibodies (1:500; Life Technologies, Carlsbad, CA) were applied for 2 hr at room temperature in the dark. DNA was labeled with Hoechst 33342 (10 μg/ml; 10 min) and coverslips mounted in SlowFade Diamond antifade mountant (Life Technologies). For ICSI-fertilized oocytes, the zona pellucida was first removed by acid Tyrode’s. Both ICSI inseminated and Sendai virus extract fused oocytes were attached to polylysine-coated coverslips before fixing in 2% paraformaldehyde in mHTF-Hepes for 30 min at 37 °C. Processing and immunostaining was accomplished as describe.

### Immunohistochemistry of intact male reproductive tracts

For immunohistochemistry, cleaned reproductive tracts (see above) were fixed, *in toto*, in 4% methanol-free paraformaldehyde (Polysciences, Warrington, PA) in mHTF-Hepes overnight at 37 °C. After fixation, reproductive tracts were washed 3x in sterile PBS, air dried at -20 °C for 2–4 hrs, and embedded in Optimal Cutting Temperature compound (Tissue-Tek OCT; VWR, Bridgeport, NJ). Embedded reproductive tracts were stored at -80 °C. For processing, 7-μm sections were cut on a cryostat at −20 °C (CM1850 UV cryostat; Leica, Buffalo Grove, IL), collected on clean glass slides (Diamond White Glass 25 × 75 × 1 mm, (+)- charged; MidSci, Valley Park, MO), and stored at −80 °C until staining. To detect direct GFP expression, slides were brought to room temperature for 5 minutes and stained for 10 min with 10-μg/ml Hoechst 33342. Sections were sealed in antifade solution using a clean coverslip before imaging. In some instances, GFP CETN2 sections were first immunostained using antibodies to Ak15 γ-tubulin (1:500; Sigma-Aldrich) and microtubules (YOL1/34; 1:200; EMD Millipore) as describe above.

### Imaging and analysis

Direct immunofluorescence of live sperm or tail-tip cells was accomplished using a epifluorescent inverted Nikon Eclipse T_i_ microscope equipped with a monochrome camera, Nikon Elements software, and differential interference contrast (DIC) objectives. Fixed slides were imaged with a Nikon A1 four-laser line confocal microscope equipped with Elements acquisition and analysis software using the same objectives. Specimen imaging was standardized for depth of focus (z-setting), box area size, and laser power settings for all four channels to facilitate comparison of spermatozoa in testicular, epididymal and vas deferens sections. For analysis, image projections were counted for the number of GFP CETN2 or centrin 20H5-tagged centrioles versus DNA sperm heads in ~100 spermatozoa per tubule, with a minimum of three tubules per section recorded. At least three mouse reproductive tracts were analyzed and all data plotted as the percentage of spermatozoa demonstrating centrioles at the position obtained in the reproductive tract. Photographic panels were prepared from selected tagged image file format (TIFF) images in Adobe Photoshop (Adobe Systems, San Jose, CA).

### Statistics

We utilized Microsoft Excel for determining mean ± standard deviations (s.d.), box plots, and statistical significance based on Student’s *t* test (two-tailed), with actual p valves expressed. Significance was determined as p < 0.5. All box plots show median (horizontal lines), 25th and 75th percentiles (small boxes), and 5th and 95th percentiles (whiskers). Graphical analyses shown are indicative of average values ± standard deviation. For all experiments, more than three trials were performed, and data are representative of all trials.

## Additional Information

**How to cite this article**: Simerly, C. *et al*. Post-Testicular Sperm Maturation: Centriole Pairs, Found in Upper Epididymis, are Destroyed Prior to Sperm’s Release at Ejaculation. *Sci. Rep.* **6**, 31816; doi: 10.1038/srep31816 (2016).

## Supplementary Material

Supplementary Information

## Figures and Tables

**Figure 1:**
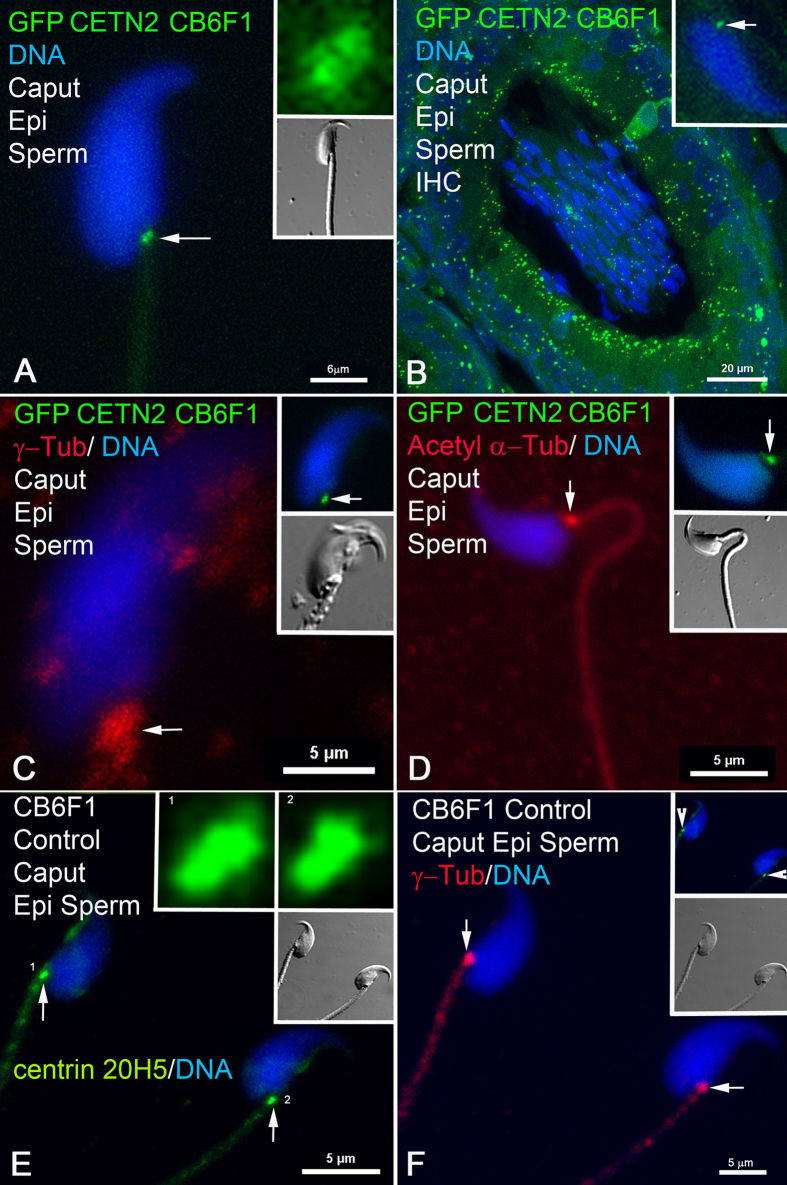
Centrin Detection in Sperm Centrioles in Caput Epididymis: GFP centrin, γ-tubulin and acetylated α-tubulin detected in control and GFP-CETN2–expressing CB6F1 sperm centrioles in caput. **(A)** In the caput (region closest to the testis), direct imaging of transgenic CB6F1 sperm expressing GFP-CETN2 demonstrates a pair of centrioles in the implantation fossa region at the base of the sperm head (green foci, arrow; DNA, blue). Top inset: higher magnification of GFP-CETN2 centrioles (green) showing adjacent positions of the proximal and distal centrioles. Lower inset: DIC. **(B)** Immunohistochemistry (IHC) cross-section of a caput epididymis tubule from a GFP-CETN2–expressing CB6F1 male. >95% of the sperm within the posterior caput lumen express GFP-CETN2 at the centrioles (green; inset: centriole detection in single sperm, arrow). Somatic cells forming the caput tubule’s lumen also express GFP-CETN2 in their basal bodies (green). **(C)** Diffuse γ-tubulin (red, arrow; blue, DNA) surrounds the GFP-CETN2–expressing centrioles in caput epididymal sperm (upper inset: green, arrow; lower inset: DIC). **(D)** Acetylated α-tubulin, a post-translationally modified tubulin, is in the sperm tail and surrounds the centriole pair (red, arrow) in a GFP-CETN2–expressing CB6F1 caput spermatozoon (upper inset: green, arrow; lower inset: DIC). **(E,F)** Non-transgenic CB6F1 caput epididymal control spermatozoa (lower insets, DIC), immunostained with monospecific antibodies to centrin 20H5 to detect the centriole pairs at the implantation fossa (E: green, arrows; upper insets: higher magnification of proximal and distal centrioles in spermatozoa 1 and 2; F: upper inset, green, arrowheads), surrounded by γ-tubulin pericentriolar material (F: red, arrows). Confocal images are direct fluorescence of expressed GFP (**A–D**) counterimaged with indirect immunofluorescence of γ-tubulin (red: **C**) or acetylated α-tubulin (red: **D**) detection or of centrin 20H5 (green: **E,F**) and γ-tubulin (red: **F**). Blue: Hoechst 33342 DNA. DIC: differential interference contrast. Bars, μm.

**Figure 2:**
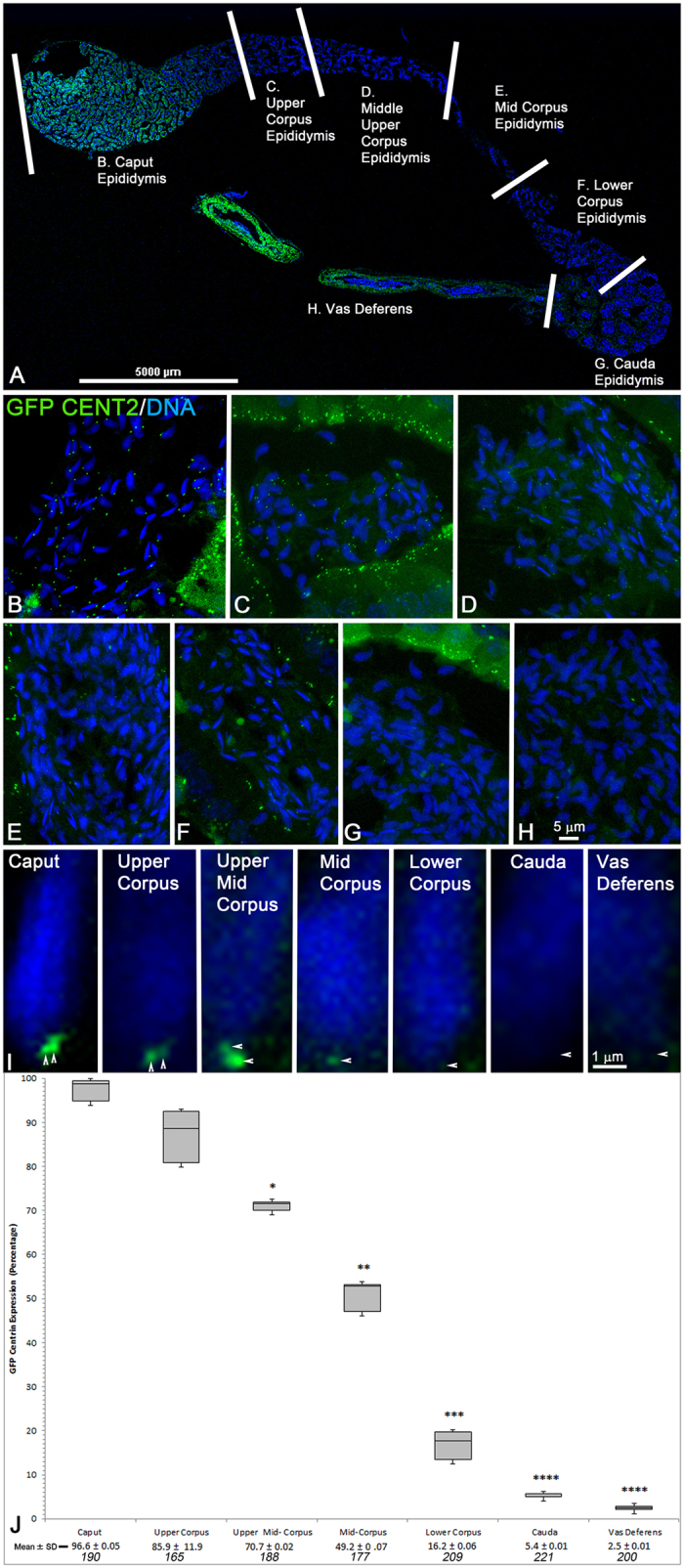
GFP Centrin Is Lost From Sperm Centrioles During Transit Through Epididymis; Centrin Detected in Only 5.4% and 2.5% of Sperm Centrioles in the Cauda Epididymis or Vas Deferens, Respectively. (**A**) Confocal stitched photomontage of GFP-CETN2 in complete epididymis with associated vas deferens. High magnification confocal images collected from tubule cross-sections from defined epididymal areas^57^ (delineated by white bars), including the caput (B), upper corpus (C), upper middle corpus (D), mid-corpus (E), lower corpus (F), cauda (**G**) and vas deferens (H). (**B**–**H**) Cross-sections of single tubules from epididymis and vas deferens in (A). GFP-CETN2 is brightly expressed in centrioles (green) from caput (**B**), upper corpus (**C**) and middle upper corpus (D) sperm, as well as luminal cell basal bodies. GFP-CETN2 signal diminishes significantly in the mid-to-lower corpus, with bright luminal GFP-CETN2 basal body expression serving as internal control (E,F: green). Spermatozoa from the cauda (G) and vas deferens (H) rarely express GFP centrin at the implantation fossa centriolar region (green) although lumen cells retain distinct basal body GFP-CETN2 expression. (**I**) Series of GFP-CETN2 expression in sperm from epididymal sections identified in (A) demonstrating the gradual dissolution of the proximal and distal centrioles at the implantation fossa during epididymal transport toward the cauda and vas deferens (green, arrowheads). (**J**) Quantification of GFP-CETN2 sperm centriole dissolution during epididymal transport as shown in (A). The box plot shows median (horizontal lines), 25th and 75th percentiles (boxes), and 5th and 95th percentiles (whiskers) of percentage of GFP-CETN2 centriole expression at the implantation fossa at each stage of transport through the epididymis. The mean ± standard deviation (SD) and total number of sperm counted (specified in italics; minimum of 3 tubules per section) is given at graph bottom. *P < 10^−3^; **P < 10^−4^; ***P < 10^−5^; ****P < 10^−6^ (Student’s t-test). All confocal imaging employed identical exposure settings (z-depth sections; 488 laser line intensity; image box size). All images: direct GFP centrin expression (green) and DNA with Hoechst 33342 (blue). Bars: μm.

**Figure 3:**
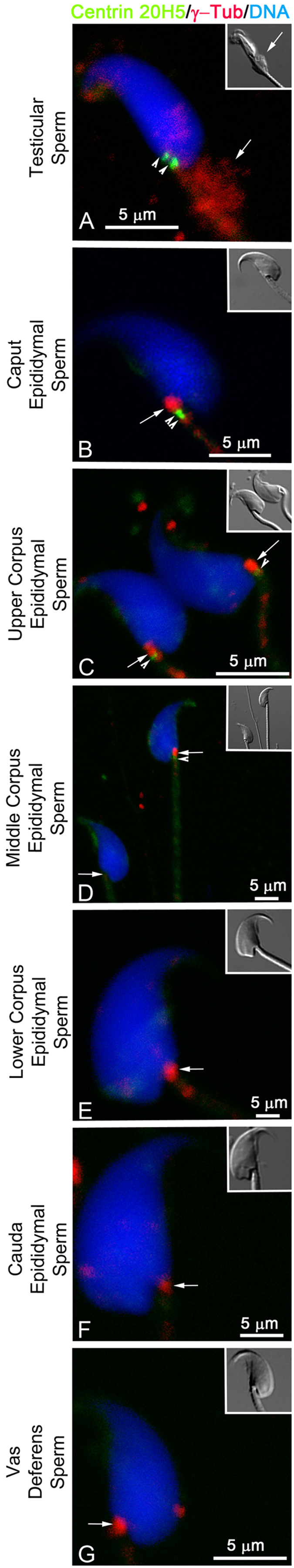
Centrin Is Lost Also in Non-Transgenic Males During Epididymal Transport: Centrin Versus γ-Tubulin in Sperm Centrioles. Testicular, epididymal and vas deferens spermatozoa immunostained with antibodies to centrin 20H5 (green, arrowheads), AK15 γ-tubulin (red, arrows) and DNA (blue). **(A)** In testicular sperm, both the proximal and distal centrioles at the implantation fossa are detected by centrin (green, arrowheads) with the majority of AK15 γ-tubulin observed in the cytoplasmic droplet attached to the upper sperm axoneme (A: red, arrow; inset: DIC) or surrounding the centriole pairs. **(B)** Caput epididymal spermatozoa demonstrate strong proximal and distal centriole detection with centrin 20H5 (green, arrowheads), while remaining γ-tubulin coalesces adjacent to the centriole pairs at the sperm axoneme’s basal body region (red, arrows). **(C)** In the upper corpus, a majority of the spermatozoa begin to show a reduction in centrin intensity at the implantation fossa (green, arrowheads), but not **γ**-tubulin (red, arrows). **(D)** In the middle corpus, spermatozoa can retain a strong **γ**-tubulin signal (upper sperm: red, arrow) and the centrin intensity in the distal centriole is frequently lost, as the proximal centriole continues to further diminish (green, arrowhead). Some spermatozoa lose both centrin and γ-tubulin detection at this stage (lower sperm: arrow). **(E–G)** In spermatozoa isolated from the lower corpus **(E)** cauda **(F)** and vas deferens **(G)** centrin no longer detects any centrioles (green) even though most sperm retain detectable γ-tubulin at the implantation fossa (red, arrows). All images are triple-labeled for centrin 20H5 (green), AK15 γ-tubulin (red) and DNA (blue). Insets: DIC: differential interference contrast. Bars: μm.

**Figure 4:**
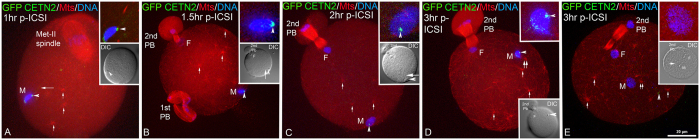
Centrioles in Sperm From the Caput Are Non-Functional Following Insemination Into Mature Zona-Intact Mouse Oocytes, Using Intracytoplasmic Sperm Injection (ICSI). (**A**) GFP-CETN2 is detected at the centriole pair at the implantation fossa (green, arrowhead) of a caput spermatozoon one hour after fertilization by ICSI. Microtubules are not detected assembling at the sperm head (red, microtubules; blue, DNA) as might be predicted if a sperm aster were to form. The sperm axoneme (red; long arrow) remains detectable, attached to the still condensed sperm head (blue). The oocyte remains arrested at metaphase-II and cytoplasmic microtubules, assembled into small cytasters, are found throughout the cytoplasm (red, short arrows). Top inset: GFP-CETN2 caput spermatozoa centrioles, green; microtubules, red; and DNA, blue. Lower inset: DIC showing sperm head (arrowhead). (**B**,**C**) By 1.5–2 hrs post-ICSI, the microinjected sperm head initiates decondensation to form the male pronucleus (M, male pronucleus; blue, DNA) as the female pronucleus forms (F, female pronucleus; blue, DNA) and 2nd polar body extrusion (2nd PB) within the oocyte’s cytoplasm was activated, probably by the microinjection event itself. The GFP-CETN2 centrioles remain at the implantation fossa (green, arrowheads) in a region devoid of assembling microtubules (red; microtubules, short arrows), even though many cytoplasmic MTOCs nucleate microtubules. Top inset: sperm decondensation (blue), GFP-CETN2 centrioles (green, arrowheads) and microtubules (red) at the sperm heads. Lower inset: DIC showing ICSI sperm (arrowheads) with attached axoneme (long arrows), female pronucleus (F) and 2nd polar body. The dividing 1st polar body is identified in B. (**D**,**E**) Two zygotes 3 hrs post-ICSI. Early male pronuclear decondensation (D: M; blue, DNA) shows caput spermatozoon GFP-CETN2 centrioles adjacent to the sperm head (D: green, arrowhead). As male pronucleus nears completion (E: M; blue, DNA), the GFP-CETN2 signal appears to disperse into punctate foci (E: green, arrowhead). Numerous microtubule asters assemble from cytoplasmic MTOCs (D, E: red; microtubules, short arrows), and one is near the male pronucleus (D, E: red, short double arrows), but it does not emanate from the GFP-CETN2 centrioles (D, E: green, arrowheads) in the activated zygotes with female pronucleus (D, E: F female pronucleus) and 2nd polar bodies. Insets: (B,C): sperm axoneme (short arrow). Triple-labeled for GFP-CETN2 centrioles (green), microtubules (red) and DNA (blue). DIC: differential interference contrast. Bar: 20 μm.

**Figure 5:**
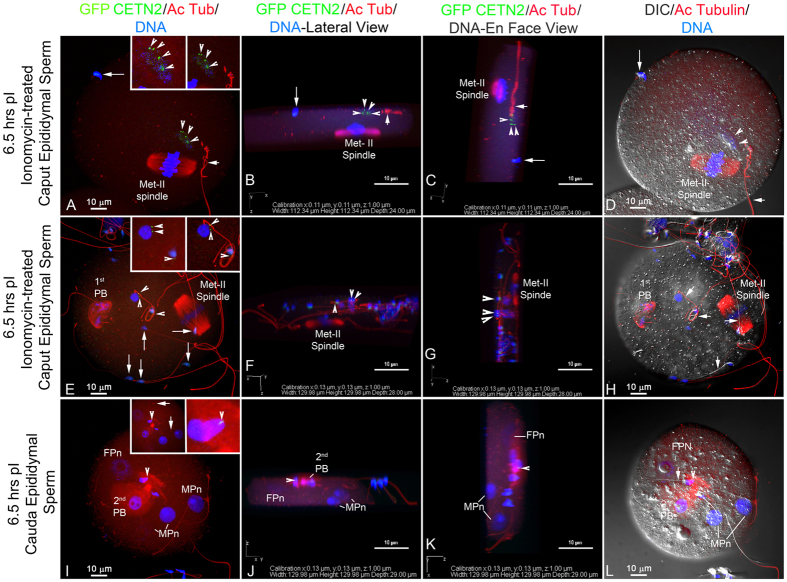
Neither Caput nor Cauda Sperm Centrioles Nucleate Microtubules Following Zona-Free IVF. **(A–H)** Zona-free oocytes fuse with immotile immature epididymal sperm in the presence of Sendai virus fusion extract, though the oocytes are typically polyspermic. The caput sperm were first treated with ionomycin to assist with membrane fusion with the zona-denuded oocytes. (**A–D)** Monospermic Insemination With Caput Sperm. E-H Dispermic Insemination With Caput Sperm. By 6.5 hrs post-insemination, two decondensing sperm heads (A, E: blue, DNA) with GFP-CETN2 centrioles are detectable (A, E green, arrowheads) though microtubules do not assemble adjacent to the incorporated sperm (A, E: left insets: red, microtubules). Oocytes remain at metaphase-II arrest (A, E: red), suggesting caput sperm, even after ionomycin, cannot initiate oocyte activation. Acetylated α-tubulin–labeled axonemes (red) are detached (A: short arrow; right inset: red) or adherent (E and right inset: red). A, E (long arrows): Bound, unincorporated sperm at the oocyte surfaces. Cauda Sperm: **(I–L)** Polyspermic Insemination With Cauda Sperm. Inseminated with cauda GFP-CETN2-sperm. By 6.5 hrs post-insemination, three decondensed male pronuclei are visible (I: MPn; blue, DNA) and 2^nd^ polar body extrusion demonstrates oocyte activation (I: FPn, female pronucleus; red). Neither GFP-CETN2 (I: green) nor microtubule assembly (I: red) near the male pronuclei are observed. (I: A rare GFP-CETN2–expressing cauda sperm early in decondensation (green, arrowheads; right inset: arrowheads; red: acetylated α-tubulin) is detected in a region devoid of assembled microtubules (left inset: red, microtubules). Maternal cytoplasmic MTOCs assemble microtubule asters (left inset: arrows). (**B,C,F,G,J,K**) rotation renderings of z-stacked image discriminate incorporated sperm (blue, arrowheads) vs unincorporated ones (long arrows), together with GFP-CETN2 centrioles (green, arrowheads) and axonemes (red, short arrows) in lateral (**B,F,J**) and *en face* (**C,G,K**; directionality, figure bottom). Sperm heads/male pronuclei ((**J**,**K**): MPn, arrowheads), female pronuclei ((**J**,**K**): FPn). Quadruple-labeled for GFP-CETN2 (green), acetylated α-tubulin (red and right insets) or YOL 134 microtubules (left insets, red), and Hoechst (blue). Bars: μm.
