# Supplementary material for: Post-Testicular Sperm Maturation: Centriole Pairs, Found in Upper Epididymis, are Destroyed Prior to Sperm’s Release at Ejaculation
Source: Sci Rep. 2016 Aug 18;6:31816. doi: 10.1038/srep31816 (PMC4989225; doi:10.1038/srep31816)
Supplement: Supplementary Information [file srep31816-s1.pdf]

SUPPLEMENTAL INFORMATION

Post-Testicular Sperm Maturation: Centriole Pairs, Found in Upper Epididymis, Are Destroyed Prior to the Sperm's Release at Ejaculation

By

C. Simerly, C. Castro, C. Hartnett, C. C. Lin, M. Sukhwani, K. Orwig,

and G. Schatten

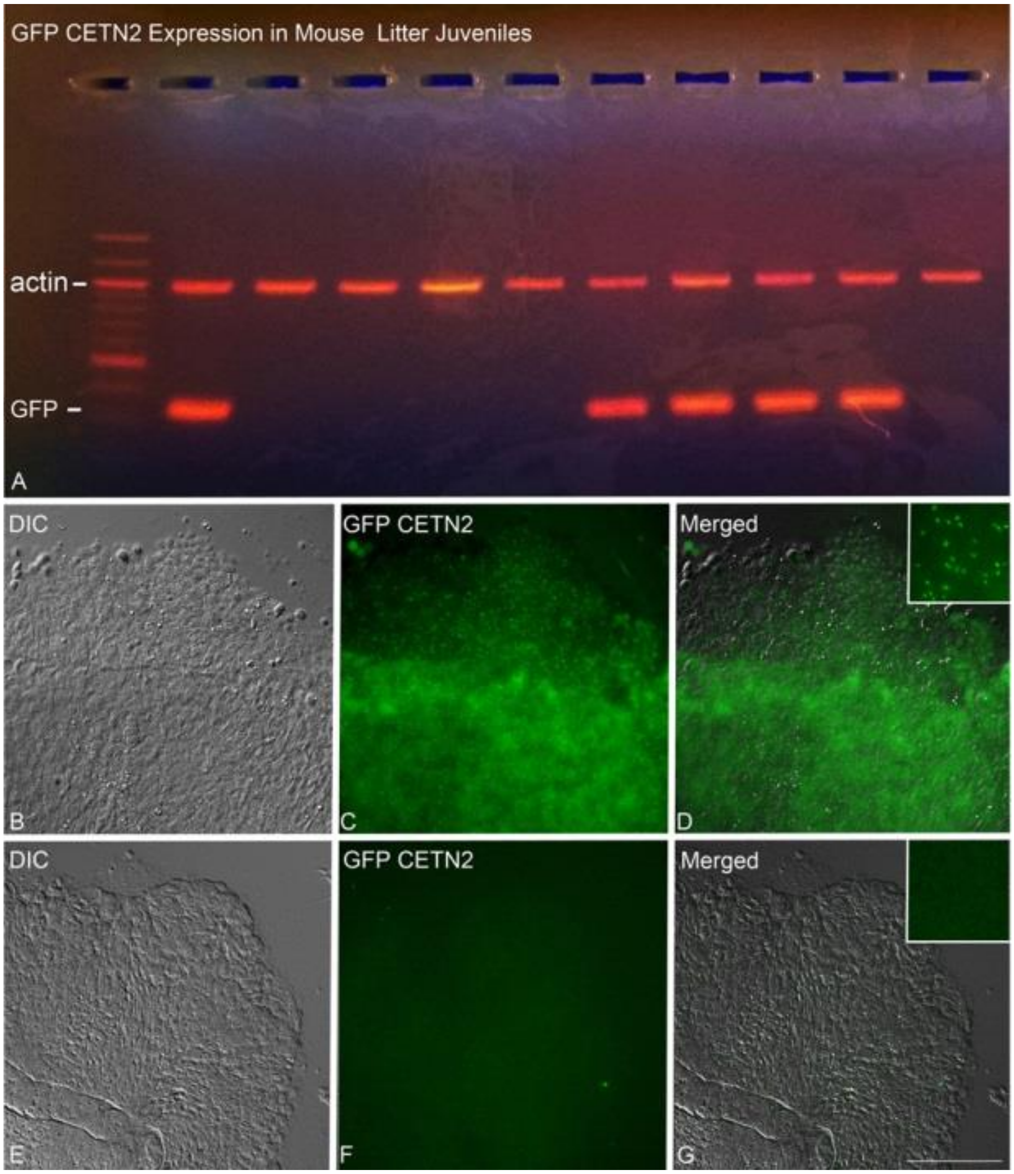

Supplemental Figure 1. GFP CETN2 expression in juvenile mice born after mating GFP CETN2 mice with non-transgenic siblings or CB6F1/J animals. (**A**): Agarose gel of PCR product produced from tail tips of a weaned litter at Day 21 post-birth, showing 5 positive GFP CETN2-expressing siblings out of 10 juvenile mice (lower bands, GFP; upper bands: actin loading control). (**B-D**): Direct fluorescent imaging of tail-tip cells (B: DIC) from a positive GFP-expressing mouse showing GFP CETN2 centrioles in the tissue (C: green). D: overlay of DIC-GFP CETN2 (inset: green, higher magnification of centrioles expressing GFP CETN2). (**E-G**): Direct fluorescent imaging of tail-tip cells (E: DIC) from a negative GFP-expressing mouse, showing no GFP CETN2 centriole detection (F: green) in this tissue. G: overlay of DIC-GFP CETN2 (inset: green, higher magnification of tissue, showing lack of detection of GFP CETN2 centrioles). Bar, 100 µm.

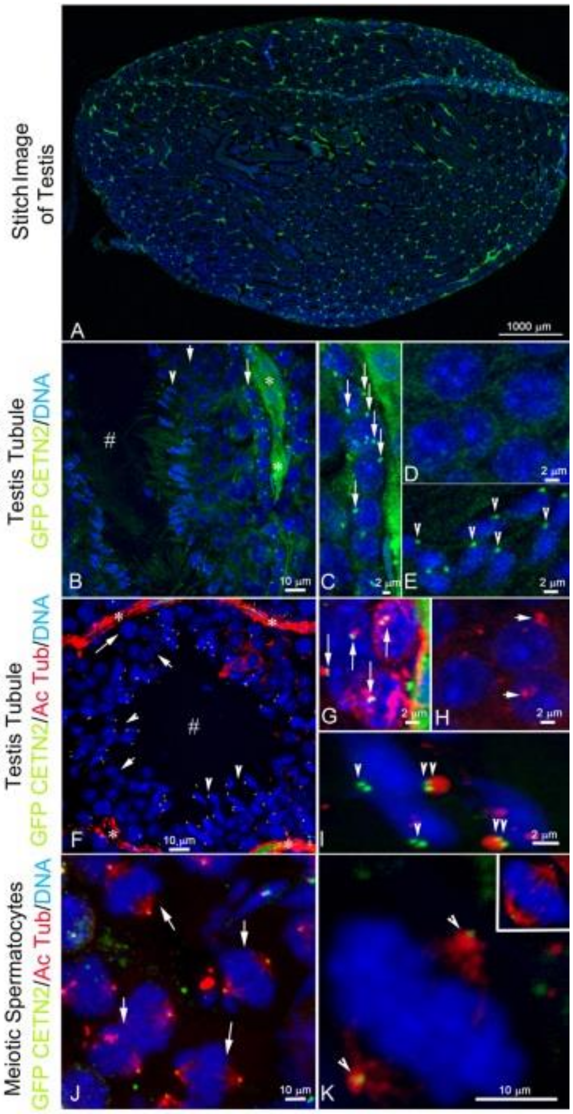

Supplemental Figure 2. GFP CETN2-expressing CB6F1 testis. (**A**): An immunohistochemistry stitch image of a GFP CETN2-expressing testis (green), showing abundant tubules in longitudinal and cross section (blue, DNA). (**B-E**): Direct expression of GFP CETN2 in a single cross section of a testis tubule from the basement membrane (B:*) to the lumen (B:#). Aligned adjacent to the basement membrane (B: long arrow, blue, DNA), spermatogonium and primary spermatocytes (C: blue, DNA) express GFP CETN2 in centrioles (C: green, long arrows) while round spermatids distal to the basement membrane (B: blue, short arrow) do not (D: green; blue, DNA). Elongated spermatids (B: blue, arrowhead) near the lumen re-express GFP CETN2 at sperm basal bodies in the implantation fossa (E: green, arrowheads; blue, DNA). (**F-I**): Cross section of a single testis tubule expressing GFP CETN2 (green) from the basement membrane (F:*) to the lumen (F:#) and co-stained with an antibody to γ-tubulin (red; blue, DNA). Spermatogonia and primary spermatocytes along the basement membrane (F: long arrow) expressing GFP CETN2 (G: green, arrows) have associated γ-tubulin adjacent to or surrounding the centrioles (G: red, arrows; blue, DNA). Round spermatids (F: short arrows) do not express GFP CETN2 (H: green) but do retain γ-tubulin at the centrosomes (H: red, short arrows; blue, DNA). A majority of elongated spermatids (F: arrowheads) expressing GFP CETN2 at the basal bodies (**I**: green, arrowheads) are associated with γ-tubulin (I: red; double arrowheads), although some advanced spermatids may shed their γ-tubulin in developing cytoplasmic droplets near the completion of spermiation (I: red, single arrowheads; blue, DNA). (**J**): A group of meiotic spermatocytes (arrows) showing expression of GFP CETN2 (green) and co-localized γ-tubulin at both spindle poles (red; blue, DNA). (**K**): Details of a meiotic metaphase spermatocyte with a bipolar spindle (inset, red, microtubules) and aligned chromosomes (inset, blue). Both poles express GFP CETN2 (green, arrowheads) with co-localized γ-tubulin (red, arrowheads). All images direct GFP CETN2 expression (green) and DNA (blue); F-K: double-labeled with γ-tubulin (red) and microtubules (red; converted from Cy5 channel). Bars, µm.

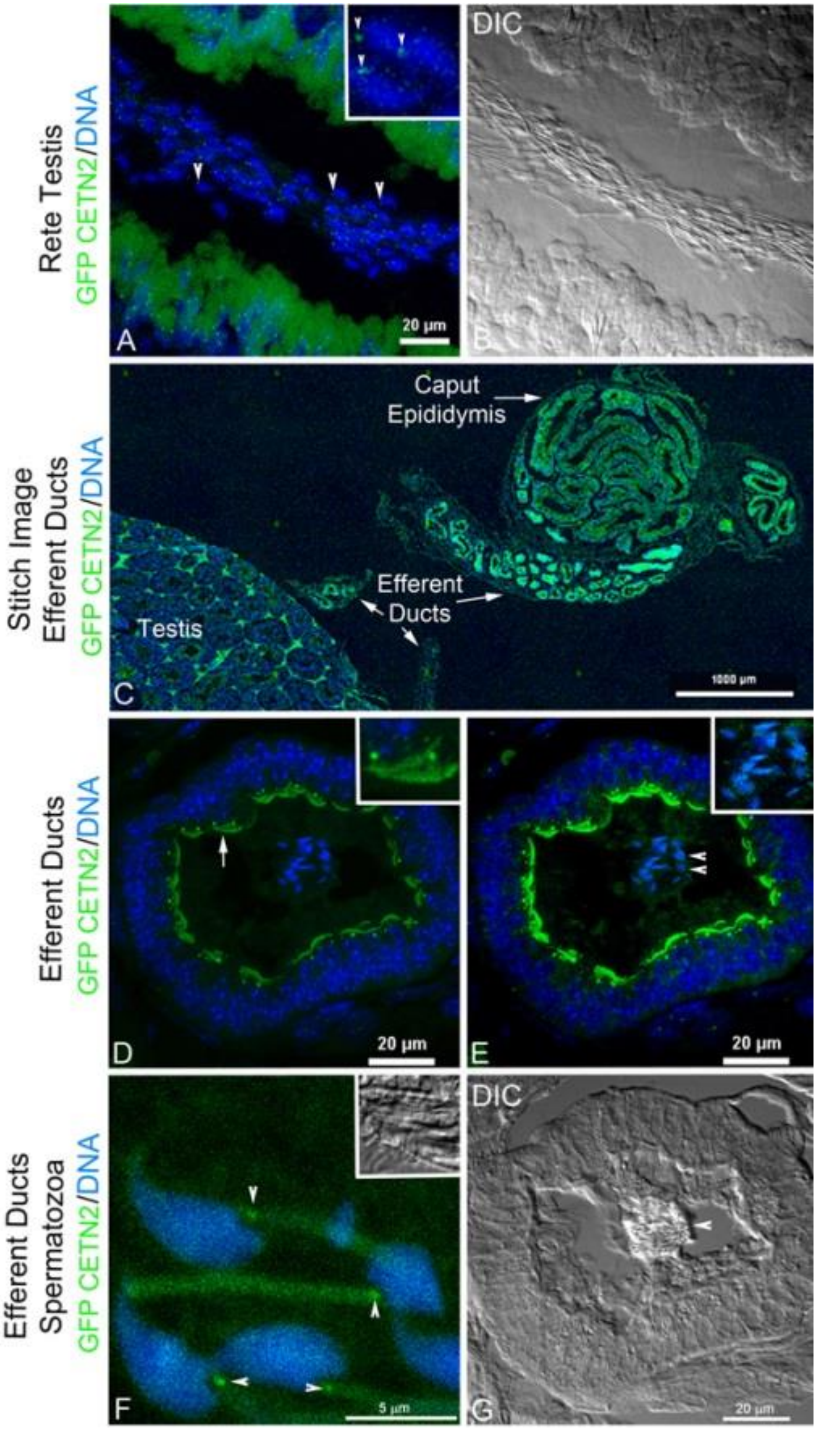

Supplemental Figure 3. GFP CETN2-expressing spermatozoa during rete testis and efferent duct transport to the epididymis. (**A-B**): Longitude section through a portion of the rete testis showing luminal transport of GFP CETN2-expressing testicular spermatozoa (A: green, arrowheads; blue, DNA; inset: GFP CETN2 details, arrowheads; B: DIC). (**C**): Stitch image of testis and efferent ducts connecting to the caput epididymis from a GFP CETN2-expressing male (green; DNA, blue). (**D-G**): Cross sections of efferent duct tubules from GFP CETN2-expressing male shown in C above. (D): Image showing ciliary basal bodies in columnar lumen cells expressing GFP CETN2 (arrow; inset: details in a single lumen cell). (E, F): Spermatozoa maintain expression of GFP CETN2 during efferent duct transport to the anterior caput epididymis (E and F: green, arrowheads; E inset: direct GFP CETN2 detection in lumen spermatozoa; F inset: DIC of spermatozoa). G: DIC image of efferent duct tubule with spermatozoa in central lumen (arrowhead). Bars, µm.

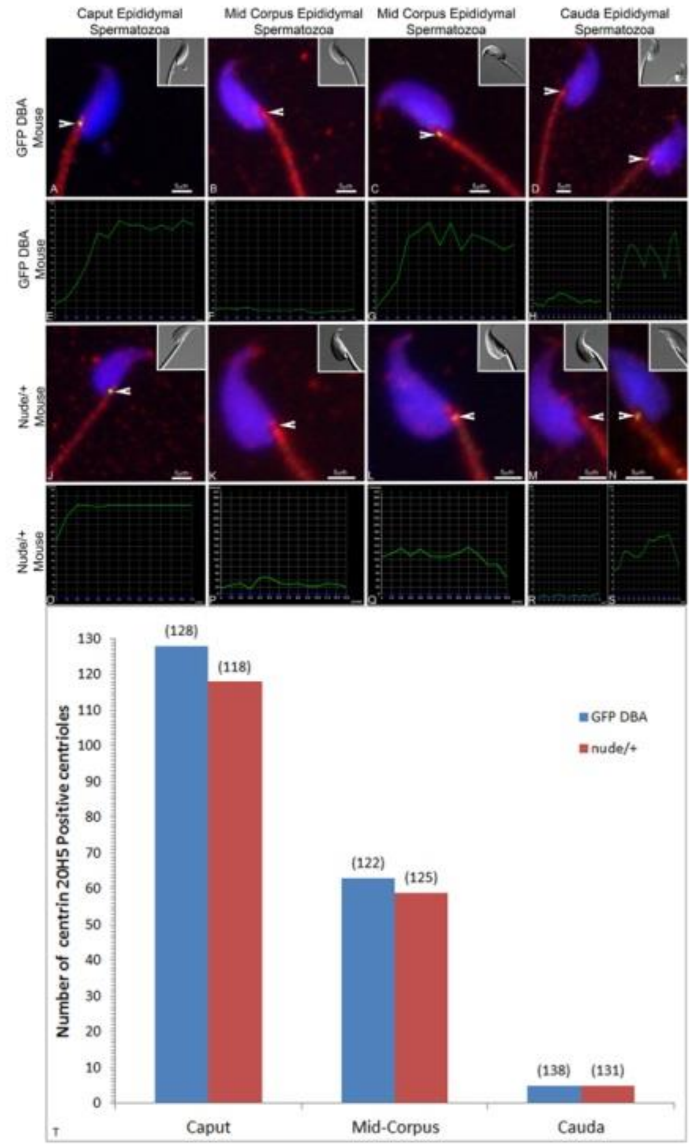

Supplemental Figure 4. Expression of centrin 20H5 in GFP-DBA and Nude/+ epididymal spermatozoa. (**A, E**): GFP-DBA mouse caput epididymal spermatozoon with centrin 20H5 detection at centrioles (A: green, arrowhead). Intensity measurement of caput epididymal spermatozoon centrioles shows strong GFP fluorescence (E). (**B,C,F,G**): Representative mid-corpus epididymal GFP-DBA spermatozoa. ~ 40% of the GFP-DBA mid-corpus spermatozoa demonstrate no centrin 20H5 detection (B: green, arrowhead) and low fluorescent intensities at the implantation fossa (F), indicating centriole dissolution. Although ~ 60% of the mid-corpus spermatozoa retain centrioles (C: green, arrowhead), these spermatozoa show diminished fluorescent intensities (G) relative to caput spermatozoa. (**D, H, I**): GFP-DBA mouse cauda epididymal sperm showing further reduction of centriolar centrin 20H5 (D: green, arrowheads). H: ~ 90% of cauda GFP-DBA sperm demonstrate nominal fluorescent intensity at the centrioles, with only~ 5% of spermatozoa showing centrioles with highly diffuse GFP (D: right sperm; green, arrowhead) and significantly reduced fluorescent intensity (I: ~ 79% less than caput sperm). (**J- N**): Nearly identical centrin 20H5 staining patterns in spermatozoa from Nude/+ mouse caput (J), mid-corpus (K, L) and cauda (M, N) epididymal sperm was observed. Fluorescent intensity measurements in centrioles of Nude/+ sperm diminished with epididymal transport (**O, P, R**), being only ~ 44% and ~52% relative to Nude/+ caput spermatozoa in mid-corpus and cauda epididymal spermatozoa (**Q, S**), respectively. This suggests a conserved pattern of centriole loss during epididymal transport among various mouse strains. (**T**): Graphic depiction of centrin 20H5 detection in GFP-DBA and Nude/+ mouse spermatozoa during epididymal transport. Images captured at same laser intensities, z-stacks, collection sizes, and LUT values for fluorescent intensity analyses, collected using Elements software and performed across 13 pixels at the mid-diameter of centrioles or the implantation fossa. (**A,B,C,D,J,K,L, M, N**): Triple-labeled for centrin 20H5 (green), microtubules (red) and DNA (blue). Bars, µm.

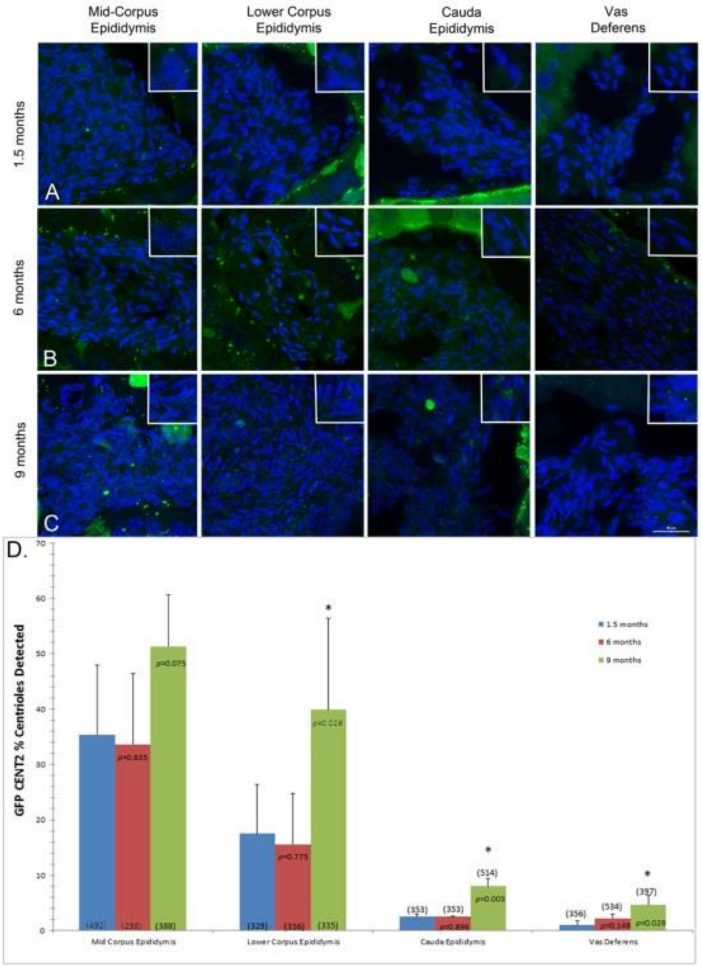

Supplemental Figure 5. **Centriole retention during aging: Oldest males retain sperm centrioles longer than younger males.** Representative images of direct GFP CETN2 detection in the epididymis (mid-corpus, lower corpus, cauda) and vas deferens tubule cross sections at 1.5 months (Row A), 6 months (Row B), and 9 months (Row C) post birth. Strong GFP CETN2 detection in sperm centrioles was observed in caput, upper corpus, and middle upper corpus tubules at all male ages examined (data not shown). Beginning at the mid-corpus, GFP centrin detection diminishes at the implantation fossa (A, B, C: green; insets, higher magnification of representative spermatozoa) in all males, but less so at 9 months of age (D: graph, green bar). Barely detected GFP centrin centrioles are found in the lower corpus epididymal tubules at 1.5- and 6-month-old males (A, B: green), with a significantly higher detection of centrin-labeled centrioles at 9 months (C: green; D: graph, green bar; ∗: significantly different from 1.5 month male). In cauda epididymis, the vast majority of spermatozoa have lost the GFP CETN2 expression at the implantation fossa (A, B: green; insets: high magnification of representative spermatozoa) at 1.5 and 6 months (D: graph, blue and red bars), while centrin detection in centrioles at 9 months remains significantly greater than in younger males (C: green, inset: details of GFP CETN2; D: green bar; ∗: significantly different from 1.5 month male). However, in the vas deferens, GFP CETN2 expression in spermatozoa at all ages is vastly reduced (A, B, C: green; insets, details; D: graph), though still statistically different at 9 months (D: graph, green bar;∗: significantly different from 1.5 month male). Collectively, males appear less efficient at centriole dissolution with advancing age. All images are direct GFP CETN2 expression (green) and DNA (blue). Graph lists percentage of GFP CETN2 detected per total DNA sperm heads counted (number in parentheses). P values reported with statistical significance equal to $p < 0.05$ (indicated by ∗ above bars). Scale bar, 10 µm.

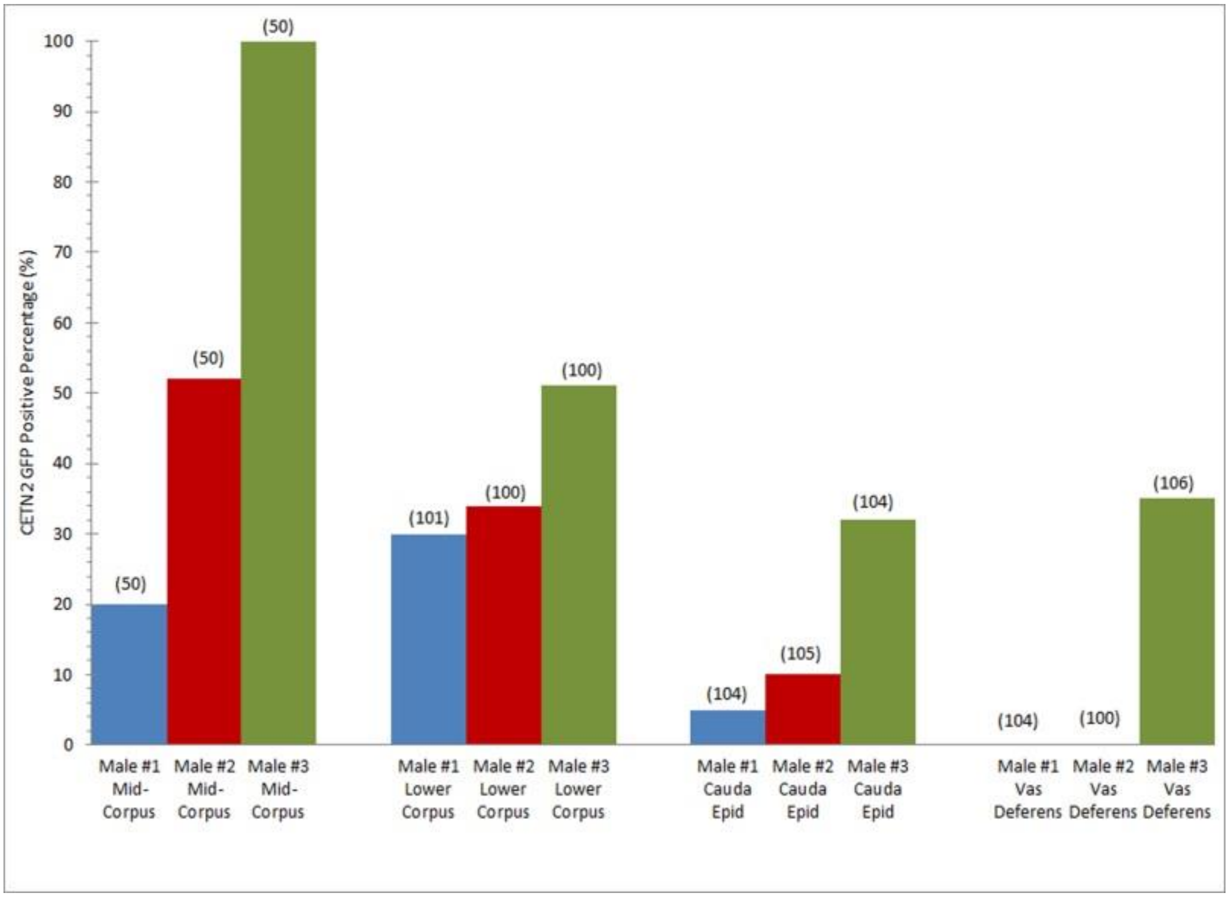

Supplemental Figure 6. Variability of centrosome detection in 15-month-old male CB6F1 CETN2. Aging males born of the same litter demonstrate variability in centriole dissolution during epididymal transport. Male 1 (blue bars) showed lower overall GFP centriole detection in the mid-corpus, lower corpus, and cauda epididymis compared to younger males (see Supp. Figure 5), although centriole detection was not observed in sperm residing in the vas deferens. Conversely, Males 2 (red bars) and 3 (green bars) demonstrated greater retention of GFP centrin detection in their centrioles at the mid-corpus and lower corpus epididymis, with Male 2 eventually losing centriole detection in the vas deferens, while Male 3 retained nearly thirty percent of the sperm with visible GFP CETN2 detection in their centrioles in the vas deferens. Graph lists percentage of GFP CETN2 detected per sperm counted (number in parentheses).
